# Fungal Communities Associated with Siricid Wood Wasps: Focus on *Sirex juvencus*, *Urocerus gigas*, and *Tremex fuscicornis*

**DOI:** 10.3390/insects15010049

**Published:** 2024-01-11

**Authors:** Adas Marčiulynas, Jūratė Lynikienė, Artūras Gedminas, Aistė Povilaitienė, Audrius Menkis

**Affiliations:** 1 Institute of Forestry, Lithuanian Research Centre for Agriculture and Forestry, Liepų Str. 1, Girionys, LT-53101 Kaunas District, Lithuania; 2 Department of Forest Mycology and Plant Pathology, Uppsala BioCenter, Swedish University of Agricultural Sciences, P.O. Box 7026, SE-75007 Uppsala, Sweden

**Keywords:** insect pests, fungal diseases, symbiosis, biological invasion

## Abstract

**Simple Summary:**

Here we investigated fungal communities associated with three species of siricid wood wasps occurring in Lithuania. Siricid wood wasps are pests of weakened forest trees or develop on the stem of dead trees, which may also vector tree-pathogenic fungi. The results demonstrated that, generally, species–specific fungal communities are associated with each species of wood wasps, and that the economically important tree pathogen *Amylostereum chailletii* was among the most common fungal taxon associated. The study, therefore, provided new insights into the ecology and symbiotic associations of these wood wasps.

**Abstract:**

We investigated the diversity and occurrence of wood wasps in Lithuania and determined communities of associated fungi. Trapping of wood wasps resulted in three different species, including *Sirex juvencus*, *Urocerus gigas*, and *Tremex fuscicornis*. Fungal culturing from adult females of *T. fuscicornis* mainly resulted in fungi from the genera *Penicillium* and *Trichoderma*. High-throughput sequencing of ITS2 rDNA resulted in 59,797 high-quality fungal sequences, representing 127 fungal OTUs. There were 93 fungal OTUs detected in *U. gigas*, 66 in *S. juvencus*, and 10 in *T. fuscicornis*. The most common fungi were *Fusarium sporotrichioides* (63.1% of all fungal sequences), *Amylostereum chailletii* (14.9%), *Penicillium crustosum* (7.8%), *Microascus* sp. 2261_4 (5.0%), and *Pithoascus ater* (2.1%). Among these, only *A. chailletii* was found in all three insect species with the highest relative abundance in *U. gigas* (15.2%), followed by *S. juvencus* (7.7%), and the lowest in *T. fuscicornis* (0.3%) (*p* < 0.0003). Correspondence analysis of fungal communities showed a distant placement of different species of wood wasps, indicating that fungal communities in each of these were largely different. In conclusion, the study showed that the economically important tree pathogen *A. chailletii* was among the most common fungal OTUs associated with siricid wood wasps.

## 1. Introduction

Wood wasps are a species of horntail insects (Siricidae), which are native to Europe, Asia, and Northern Africa [1]. Among different species of wood wasps, *Sirex noctilio* is an invasive species in Australia, New Zealand, North America, South America, and South Africa, where it causes massive dieback of pine trees and large economic losses [2]. Siricid wood wasps are secondary pests attacking only wounded, stressed, or newly felled trees [2,3]. Still, they can also infest living trees [3]. They are also known to vector tree-pathogenic fungi of the genus *Amylostereum*, which also cause serious damage to trees and wood [4]. In all investigated species of wood wasps, the adult females possess a specialized gland containing mucus and fungal oidia, which are inoculated under the bark during the oviposition. *Amylostereum* fungi start to grow in the oviposition chamber and in larval tunnels, where they colonize and degrade cellulose and wood tissues [4,5,6,7,8]. The main advantage of the fungus is that wood wasps spread and effectively inoculate new wood with the fungal material during egg laying [9,10]. At the same time, the fungal growth results in wood rot and the drying out of wood, providing the right conditions, nutrients, and enzymes that are needed for the survival and development of larvae of these wood wasps [11]. Wood wasps create optimal conditions for fungal infestation by drilling under the bark and weakening the host tree as wood wasps are fully dependent on the fungus for nutrition [12]. The decomposition of wood by the fungus results in a white rot and enables larvae to access and utilize it for food, while the lack of such wood decomposition arrests the development of larvae.

Emerged adults vector the same fungal genotype and infect new trees, which leads to the genetic diversity of the fungus being relatively low. For example, in the southern hemisphere, *S. noctilio* carries a single genotype of *Amylostereum areolatum* [1]. The siricid wood wasps and *A. areolatum* have a mutualistic relationship, thereby benefiting each other. In Lithuania, species of wood wasps that cause economic damage to conifer trees include *Sirex juvencus* and *Urocerus gigas* [13]. It was shown that *S. juvencus* vectors *A. areolatum* of large dispersive clones, which encompass several countries (i.e., the same genotypes were recovered from Lithuania, Sweden, and Denmark), and have low genetic variation between different clones [14]. *Urocerus gigas* was shown to vector *Amylostereum chailletii*, but differently from the *S. juvencus* and *A. areolatum* association, the population structure of *A. chailletii* is of high genetic diversity, indicating that the fungus often spreads via airborne basidiospores produced by outcrossing [14], thereby highlighting the differences between both insects as well as fungal species. Furthermore, *Sirex* insects often develop and oviposit with various curculionids, cerambycides, and other wood-associated insects that also colonize weakened and/or stressed trees [15]. These insects are well-known as vectors of different fungal species, primarily ophiostomatoid ones. All these beetles that usually live together with siricides transmit fungi such as *Leptographium wingfieldii*, *Leptographium lungbergii*, *Ophiostoma minus*, *Sphaeropsis sapinea*, *Ophiostoma canum*, or *Ophiostoma bicolor* [16,17,18]. Often, the fungi remain in the wood for a long time and developed insects can carry these pathogenic fungi and infect new trees.

In Lithuania, most studies on *Amylostereum* fungi are based on fungal culturing from the wood, while the information on the overall fungal community associated with wood wasps is scarce. Furthermore, it is likely that due to climate change and human economic activities (e.g., international trade) new pathogens of forest trees could spread in association with wood wasps. To understand the current situation and possible future consequences to forest health and associated biodiversity, better knowledge is needed on symbiosis between wood wasps and associated fungal communities.

The aim of the present study was to investigate the diversity and occurrence of wood wasps in Lithuania and use fungal culturing and high-throughput sequencing to determine communities of associated fungi.

## 2. Materials and Methods

### 2.1. Study Sites and Sampling

Sampling of wood wasps was carried out between June and August 2013 at three different sites in Lithuania. The study sites were coniferous forest stands characterized by a large number of weakened and declining trees, and therefore, potentially suitable for wood wasps. The study sites were situated in the Kaunas, Alytus, and Jonava districts in Central Lithuania, up to 30 km away from each other. The sampling of insects was carried out using two methods: (i) active trapping using an entomological net during the time of active flying; and (ii) extraction from the colonized wood in the laboratory [19,20,21]. For sampling from the wood, logs of Norway spruce (*Picea abies*) and silver birch (*Betula pendula*) with characteristic exit holes of wood wasps were transported to the laboratory and stored in ventilated incubation chambers at ca. 20 °C [20]. Individual insects of wood wasps that were emerging from wood logs were trapped, placed individually into 1.5 mL centrifugation tubes, and stored at −20 °C until they were used for direct extraction of DNA and sequencing of fungal communities.

### 2.2. Fungal Culturing from Wood Wasps

Fungal culturing was carried out on 30 adult females of *Tremex fuscicornis* as described by Thomsen and Hardini [21]. Briefly, insects were euthanized using chloroform, the last segment of their stomach was cut using sterile scissors under the dissection microscope, and a part with eggs and fungal material was separated. The extracted material was aseptically plated onto Petri dishes with a potato dextrose medium (PDA) [22]. The inoculated dishes were kept at ca. 20 °C in daylight conditions. The outgrowing fungi were immediately transferred to new Petri dishes with PDA, divided into different mycelial groups, and representative cultures were identified based on the morphology of fungal mycelia using a dissection microscope (Stemi 2000, CarlZeiss, Oberkochen, Germany).

### 2.3. DNA Extraction, Amplification, and Sequencing

For the identification of fungal communities, wood wasps of *S. juvencus*, *U. gigas*, and *T. fuscicornis* (Table 1), which were sampled during field trapping and/or extracted from the wood logs, were used for DNA work. Genomic DNA was isolated separately from 17 adult females of *S. juvencus*, 19 adult females of *U. gigas*, and 24 larvae of *T. fuscicornis*. Before the extraction of DNA, insects were individually placed in 15 mL centrifugation tubes and lyophilized at −60 °C for two days. Surface sterilization was not applied. Lyophilized insects were divided into smaller segments, individually placed into 2 mL screw-cap tubes together with four glass beads (2 mm in diameter), and homogenized using a FastPrep instrument (Precellys 24; BertinTechnologies, Rockville, MD, USA). DNA extraction was completed using the 3% CTAB protocol as in Menkis et al. [23].

The amplification by PCR of DNA samples was achieved using primers fITS9 (5′-GAACGCAGCRAAIIGYGA-3′) [24] and ITS4 (5′-TCCTCCGCTTATTGATATGC-3′) [25] containing 8 bp unique sample identification barcodes for sample tracking. The PCR reactions, which were 50 μL in volume for each sample, were run on an Applied Biosystems 2720 Thermal Cycler (Applied Biosystems, Carlsbad, CA, USA) using DreamTaq Green DNA polymerase (Thermo Fisher Scientific, Waltham, MA, USA). The PCR cycling parameters were as follows: initial denaturation at 95 °C for 2 min, 27 cycles of denaturation at 95 °C for 30 s, annealing at 55 °C for 30 s and extension at 72 °C for 45 s, and then a final extension at 72 °C for 7 min. The PCR products were checked using 1.5% agarose gels (Agarose D1, Conda, Madrid, Spain). Amplified products were purified using the QIAquick Gel Extraction Kit (Qiagen, Hilden, Germany), and their concentrations were determined using the Quant-iT™ dsDNA HS Assay Kit (Life Technologies, Carlsbad, CA, USA). Samples were pooled in an equimolar mix and sequenced on an Ion Torrent platform and a 316 chip. Sequencing was completed by NGI SciLifeLab (Uppsala, Sweden).

### 2.4. Bioinformatics

Bioinformatics (quality control and clustering) were carried out using the Sequence Clustering and Analysis of Tagged Amplicons (SCATA) tool available at http://scata.mykopat.slu.se, accessed on 15 August 2015. Quality control included the removal of <200 bp sequences, Q < 20 quality sequences, and primer dimers and homopolymers by collapsing them to 3 bp before clustering. Sequences lacking a barcode or primer sequence were removed. Primers and sample barcodes were then removed from sequences, storing the information on the association between sequences and samples as meta-data. High-quality sequences were assembled into different OTUs using single-linkage clustering based on 98.5% similarity. The most frequent genotype (real read) for each cluster was used to represent each OTU. A consensus sequence was produced for clusters with two sequences. The OTUs were taxonomically identified using the GenBank (NCBI) database and the BLASTn algorithm using the following criteria: sequence coverage > 80%; similarity to species level 98–100%, similarity to genus level 94–97%. Sequences deviating from these criteria remained unidentified and were given unique names as shown in Table 2. Representative sequences were submitted to GenBank and are available under accession numbers OR982194–OR982320.

### 2.5. Statistical Analyses

A non-parametric chi-square test was used to assess differences in the relative abundance of common fungal OTUs associated with different species of wood wasps [26]. In the case of multiple comparisons, confidence limits for *p*-values of the chi-square tests were divided by the number of such comparisons as required by the Bonferroni correction [27]. The diversity and composition of fungal communities associated with different species of wood wasps were characterized using the Shannon diversity index and qualitative Sørensen similarity index [26,28,29]. Correspondence analysis (CA) was done with the CA function from the FactoMineR package based on the log+1 transformed matrix of found species. To test differences in fungal communities associated with different hosts, we used the Kruskal test, with the kruskal.test function from the stats package. A heatmap was built based on a log+1 transformed matrix using the 30 most common fungal OTUs using the pheatmap function from the pheatmap package. A log transformation was applied to the selected data, adding one to each value to handle potential zero values. Correlation analysis with a Pearson correlation coefficient was performed on the log-transformed data with the cor.mat function from the rstatix package. These analyses were performed using Vegan 2.5.7 and Stats 3.6.2 in R 4.1.1 (https://www.r-project.org (accessed on 14 August 2023) [30].

## 3. Results

Sampling using an entomological net resulted in two adult females of *S. juvencus* and eight of *U. gigas*. Sampling using wood logs resulted in 15 additional wood wasps of *S. juvencus* and 11 of *U. gigas*, thereby yielding a total of 17 and 19 individuals of each species, respectively (Table 1). All wood wasps of *T. fuscicornis* sampled in the present study (30 adults were used for fungal culturing and 24 larvae for sequencing) were collected from birch logs. Fungal culturing from adult females of *T. fuscicornis* resulted in 58 pure cultures, which, following morphological assessments, were divided into 12 morphological groups. The morphological assessment revealed that the most often cultured fungi were from the genera *Penicillium* (65.5% of all cultures), *Trichoderma* (13.8%), *Fusarium* (10.1%), *Amylostereum* (5.6%), and *Scopulariopsis* (3.3%). A single culture of the ascomycete *Daldinia decipiens* (1.7%) was also detected. 

**Figure 1:**
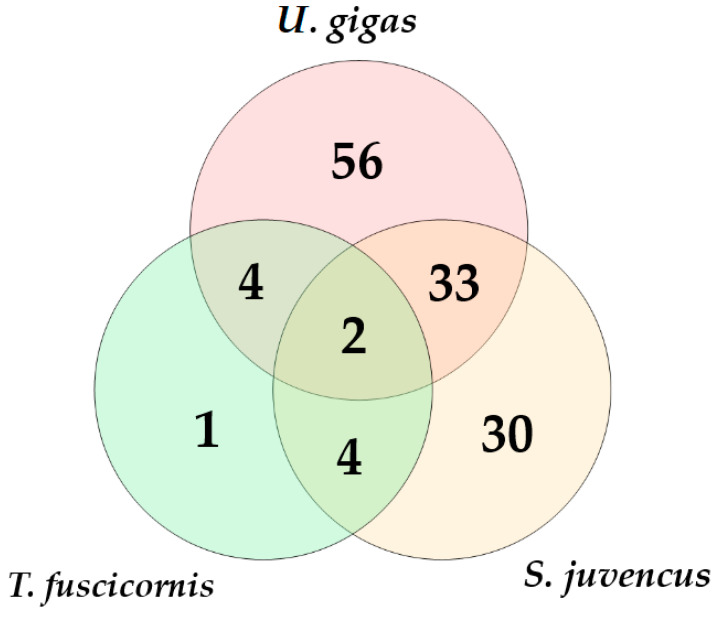
Venn diagrams showing the diversity and overlap of fungal OTUs associated with *U. gigas*, *T. fuscicornis*, and *S. juvencus*. The data from different individuals of the same insect species are combined.

Ion Torrent sequencing of ITS2 rDNA from 17 *S. juvencus*, 19 *U. gigas*, and 24 *T. fuscicornis* wood wasps resulted in 61,839 high-quality reads. The clustering of these reads showed the presence of 147 non-singleton contigs (representing different OTUs) and 210 singletons, which were removed from further analysis. Taxonomic classification showed that 127 OTUs (represented by 59,797 high-quality reads) were fungal and 20 were non-fungal, which were removed (Table 1). The distribution of fungi into different phyla was: 84.9% Ascomycota, 15.1% Basidiomycota, and 0.03% Mucoromycota. Although the absolute richness of fungal OTUs was highest in *U. gigas* (Table 1), when the number of sequences was taken into consideration, the detected richness of fungal OTUs was significantly higher in *S. juvencus* (66 OTU out of 1390 sequences) and *T. fuscicornis* (10 OTU out of 293 sequences) than in *U. gigas* (93 OTU out of 58,543 sequences) (*p* < 0.0001). The detected richness of fungal OTUs between *S. juvencus* and *T. fuscicornis* did not differ significantly (*p* > 0.05). The number of unique fungal OTUs associated with different insect species was between 1 and 56 (Figure 1). There were 33 fungal OTUs shared between *U. gigas* and *S. juvencus*, while only 4 fungal OTUs were shared between *T. fuscicornis* and *U. gigas*, and 4 between *T. fuscicornis* and *S. juvencus*. Only two fungal OTUs were common to all insect species (Figure 1).

Table 2 shows the 20 most common fungal OTUs representing 98.5% of all fungal sequences. The most common fungi were *Fusarium sporotrichioides* (63.1% of all fungal sequences), *Amylostereum chailletii* (14.9%), *Penicillium crustosum* (7.8%), *Microascus* sp. 2261_4 (5.0%), and *Pithoascus ater* (2.1%). Among all fungal OTUs, only *A. chailletii* and Unidentified sp. 5671_47 were associated with all three species of wood wasps. The abundance of *A. chailletii* was highest in *U. gigas* (15.2%), followed by *S. juvencus* (7.7%), and lowest in *T. fuscicornis* (0.34%) (difference in chi-square test significant at *p* < 0.0003). The remaining 107 fungal OTUs were very rare, and their relative abundances varied between 0.003% and 0.09% (Table 2 and Table S1).

**Figure 2:**
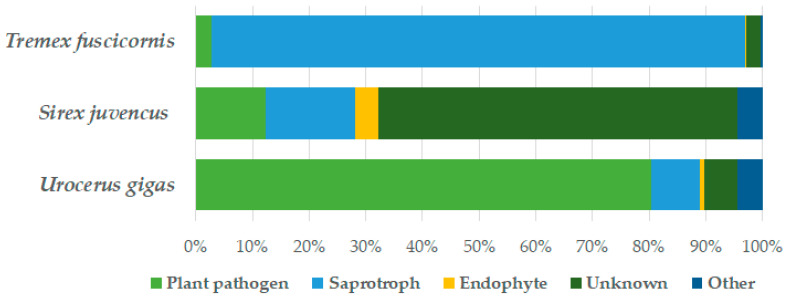
Relative abundance (%) of fungal functional groups associated with *U. gigas*, *T. fuscicornis*, and *S. juvencus*, estimated based on fungal sequences. Others represent fungi, which are not associated with plants (e.g., animal pathogens).

The determination of fungal functional groups was possible for 61 fungal OTUs representing 93.0% of fungal sequences, and Figure 2 shows their relative abundance in different insect species. Among all samples, the most common fungal functional groups were plant pathogens (78.5%) and saprotrophs (9.5%) (Figure 2). The most common plant pathogens associated with *U. gigas* were *F. sporotrichioides* (64.9%), *A. chailletii* (15.1%), *Fusarium* sp. 5671_18 (0.12%), and *Fusarium* sp. 5671_22 (0.10%); those associated with *S. juvencus* were *A. chailletii* (7.7%), *Diplodia sapinea* (1.3%), and *Alternaria* sp. 5671_28 (0.8%); and those associated with *T. fuscicornis* were *Ophiostoma* sp. 5671_103 (1.4%), *Cosmospora viridescens* and *F. sporotrichioides* (0.3%), and *A. chailletii* (0.3%). The relative abundance of plant pathogens was 80.4% in *U. gigas*, 12.4% in *S. juvencus*, and 2.7% in *T. fuscicornis*, but their relative abundances differed significantly only between *T. fuscicornis* and *U. gigas* (*p* < 0.05). The relative abundance of saprotrophs was 94.2% in *T. fuscicornis*, 15.7% in *S. juvencus*, and 8.6% in *U. gigas*. Statistically significant differences were between *T. fuscicornis* and *S. juvencus* and between *T. fuscicornis* and *U. gigas* (*p* < 0.05) (Figure 2). The relative abundance of endophytes was highest in *S. juvencus* (4.2%), which was significantly higher than in *U. gigas* (0.8%) or *T. fuscicornis* (0.4%) (*p* < 0.05) (Figure 2).

**Figure 3:**
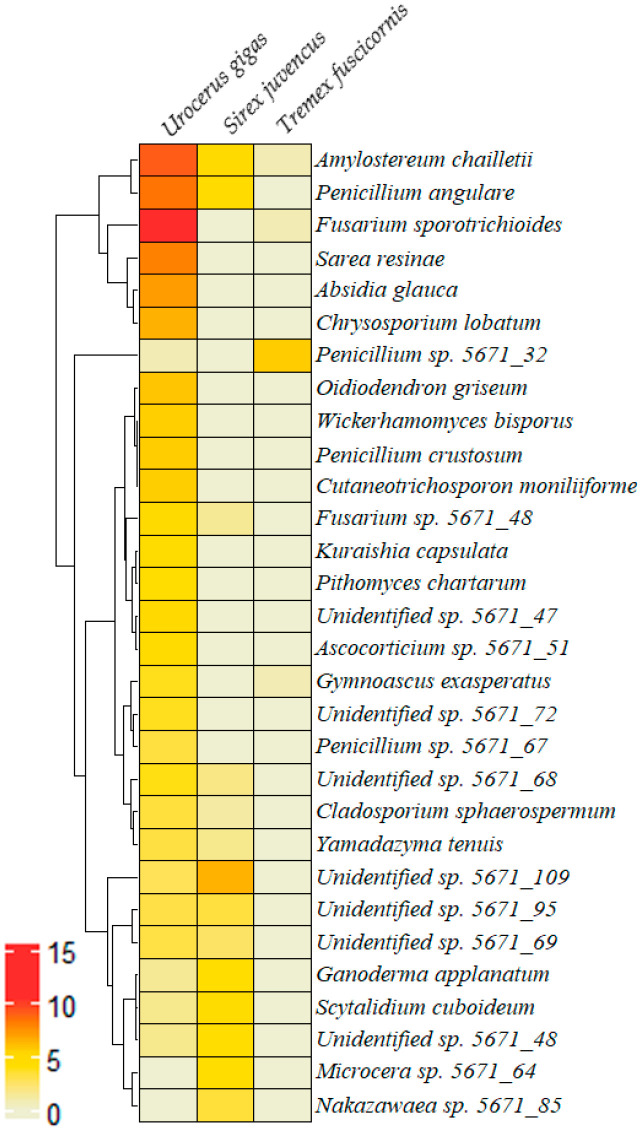
A heatmap with dendrograms of hierarchical clustering of the 30 most abundant fungal OTUs associated with different species of wood wasps. The scale shows log-transformed relative abundance.

A heatmap with hierarchical clustering of the 30 most abundant fungal OTUs showed a relatively weak association between different insect species and different fungal OTUs (Figure 3). The analysis also revealed a certain specificity of particular fungal OTUs and different species of wood wasps colonizing the wood of either conifer (*U. gigas* and *S. juvencus*) or deciduous (*T. fuxcicornis*) trees. Consequently, *F. sporotrichioides*, *A. chailletii*, and *P. angulare* showed a higher relative abundance in association with *U. gigas*, Unidentified sp. 5671_109, *A. chailletii*, and *P. angulare* with *S. juvencus*, while *Penicillium* sp. 5671_32 was associated with *T. fuxcicornis*. Strong associations among some fungal OTUs were also identified. A strong correlation was detected between *A. chailletii* and *P. angulare* (*r* = 1, *p* < 0.05), and between *Absidia glauca* and *Chrysosporium lobatum* (*r* = 1, *p* < 0.05).

**Figure 4:**
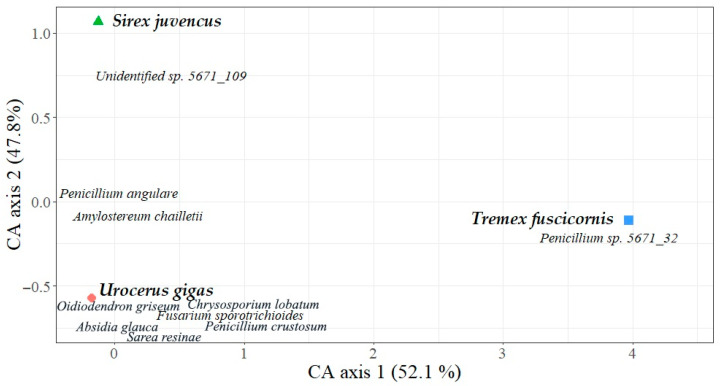
Ordination diagram based on correspondence analysis of fungal communities sequenced from three species (*Sirex juvencus*, *Urocerus gigas*, and *Tremex fascicornis*) of wood wasps sampled in Lithuania. The ten most common fungal OTUs are presented, and their positions correspond to location in the ordination (centered).

Correspondence analysis (CA) of fungal communities explained 52.1% variation on Axis 1 and 47.8% on Axis 2. CA showed that the placement of different species of wood wasps was separated along Axis 1 or Axis 2, indicating that fungal communities in each of these insects were relatively different (Figure 4). Among the ten most common fungal OTUs shown in the ordination, the majority of fungi were linked to *U. gigas*, while a single fungal OTU was linked to *S. juvencus* or *T. fuscicornis* (Figure 4). The Sørensen similarity index of fungal communities was 0.42 between *S. juvencus* and *U. gigas*, 0.12 between *S. juvencus* and *T. fuscicornis*, and 0.13 between *U. gigas* and *T. fuscicornis*. The Shannon diversity index of fungal communities was 2.4 in *S. juvencus*, 1.3 in *U. gigas*, and 0.3 in *T. fuscicornis* (Table 1).

## 4. Discussion

The sampling of wood wasps demonstrated that the methods used in the present study resulted in a relatively low number of trapped insects (Table 1). Although the trapping sites represented characteristic habitats of wood wasps, the results may suggest that the abundance of wood wasps at the time of trapping was likely low. Although the reason for this is unknown, the possibility should not be excluded that natural enemies (parasitoids) of wood wasps were responsible for keeping their population at relatively low levels [31]. In support, the deployment of parasitic nematodes in biocontrol was shown to have a significant effect on the population density and viability of *S. noctilio* [32]; however, in our study, no biocontrol methods were applied. 

The results revealed that wood wasps are associated with a high number of different fungal OTUs, including tree pathogens such as *A. chailletii*. Although *A. chailletii* was found in association with all three species of wood wasps, its relative abundance varied substantially among different insect species (Table 2). The latter may suggest that *U. gigas* and *S. juvencus* are principal vectors of this pathogen, while the association with larvae of *T. fuscicornis* appears to be occasional (Figure 3). In agreement, it was shown that *Amylostereum* spp. is most often associated with species from genera *Sirex*, *Urocerus*, and *Xoanon*, and it is classified as an insect-associated symbiotic fungus [15,33]. Infection of wood by *Amylostereum* spp. has two important functions for wood wasps: The white rot softens the wood, and at the same time the mycelium of the fungus serves as a food for the larvae [34]. As shown by the low values of the Sørensen similarity index and CA analysis, the overall fungal community structure also differed among different species of wood wasps (Figure 4), showing a certain specificity and adaptation to each particular insect species.

Similarly, fungal functional groups also differed among different species of wood wasps (Figure 2). Consequently, pathogenic fungi were most commonly associated with *U. gigas*, and saprotrophs with *T. fuscicornis*, while unidentified fungi were associated with *S. juvencus* (Figure 2). This is consistent with the previous observations as *U. gigas* and *S. juvencus* are the vectors of pathogenic *Amylostereum* fungi, while *T. fuscicornis* is known as a secondary pest that develops in already dead wood. Therefore, a high relative abundance of saprotrophic fungi in association with *T. fuscicornis* can be owing to the fact that its larvae develop in birch wood already colonized by wood decomposers [35,36].

Among the other most common fungi, several OTUs of *Fusarium* were detected (Table 2). Fungi from the genus *Fusarium* are ubiquitous in different environments [37,38] and include economically important plant pathogens, which may cause tree diseases in both managed and natural forest ecosystems [39,40]. For example, *F. sporotrichioides* is a soil-borne pathogen, which is often observed in cold climate zones such as in Northern Japan, Northern USA, or Northern Europe. Apart from agricultural crops, it was shown to be associated with seedling root rot in forest nurseries [41,42]. It was also detected in needles and branches of *Pinus* spp. trees. This pathogen was also reported in association with bark beetles in *Pinus radiata* plantations. Although it is a widespread pathogen, no association between *F. sporotrichioides* and wood wasps was reported before. Interestingly, a high relative abundance of this fungus was found only in association with *U. gigas*, but not in association with *S. juvencus* or *T. fuscicornis*. Therefore, the possibility should not be excluded that the observed association between *U. gigas* and *F. sporotrichioides* is not accidental. Indeed, previous reports and an abundant detection of *F. sporotrichioides* in association with wood wasps may suggest that the fungus can be involved in the infestation process of host trees [43,44].

Among other pathogens, *Diplodia sapinea* (0.03%) and *Ophiostoma novo-ulmi* (0.02%) were also detected at a low relative abundance in association with *U. gigas* and *S. juvencus*. *D. sapinea* is known as a common disease-causing agent of conifers, especially pines [45,46]. *O. novo-ulmi* is commonly associated with bark beetles (Scolytidae) and is the agent of Dutch elm disease of *Ulmus* spp. trees [47,48]. Although the relative abundance of this fungus was low, siricid wood wasps appear to be occasionally associated with this important pathogen of elm trees. *Daldinia decipes* is known as a symbiont of the siricids *Xiphydria camelus* and *Xiphydria longicollis*, which were isolated from the mycangia of adult females [20]. The species was previously reported to cause infections in different species of deciduous trees such as from the genera *Alnus*, *Betula*, *Quercus*, *Ulmus*, *Populus*, and *Prunus* [19,20,49]. Apart from its association with insects, this species may also produce fruiting bodies on weakened hosts, and then spread via ascospores [50]. The low relative abundance of *D. decipiens* (0.002%) in association with adults of *U. gigas* and the complete absence in association with *S. juvencus* can be due to the fact that this fungus is found on deciduous trees such as birch, alder, and oak [51,52], and/or that it has a low dependence on these vectors. By contrast, a high relative abundance of *D. decipiens* (94.54%) in association with larvae of *T. fuscicornis* can probably be due to their direct contact with fungal mycelia in colonized host trees [19]. Several species of yeasts (e.g., *Kuraishia molischiana* (0.42%), *Wickerhamomyces bisporus* (0.22%)) were also detected, which were previously shown to be associated with different bark beetles such as *Ips typographus* or *Dendroctonus* sp. [53,54,55,56].

## 5. Conclusions

The study provided new insights into the ecology and symbiotic associations of three species of wood wasps occurring in Lithuania. Moreover, the study demonstrated that, generally, species–specific fungal communities associate with each species of wood wasps investigated, and that the economically important tree pathogen *A. chailletii* was among the most common fungal OTUs associated with the wasp species analyzed in the study.

## Figures and Tables

**Table 1:** Insect materials used for ITS2 rDNA sequencing generated high-quality sequences (assembled) and detected a diversity of fungal OTUs.

Species	Stage	No. of Individuals	No. of Reads	Average read Length, bp	No. of Fungal OTUs	Shannon Diversity Index
*Sirex juvencus*	Adult female	17	1390	327	66	2.42
*Urocerus gigas*	Adult female	19	58,543	323	93	1.28
*Tremex fuscicornis*	Larvae	24	293	338	10	0.32
All		60	59,797	330	127	

**Table 2:** Relative abundance of the 20 most common fungal OTUs directly sequences from 3 species of siricid wood wasps sampled in Lithuania.

Phylum *	Species	GenBank Reference	Compared, bp; Similarity, %	*Urocerus gigas*, %	*Sirex juvencus*, %	*Tremex fuscicornis*, %	All, %
A	*Fusarium sporotrichioides*	MN452643	303/306 (99)	64.92	-	-	63.11
B	*Amylostereum chailletii*	MH857239	336/343 (98)	15.17	7.69	0.34	14.92
A	*Penicillium crustosum*	MT316358	319/323 (99)	7.84	6.48	-	7.77
A	*Microascus* sp. 5671_3	NR_155397	324/329 (98)	5.10	-	-	4.96
A	*Pithoascus ater*	NR_132951	328/333 (98)	2.14	-	-	2.08
A	*Cyberlindnera* sp. 5671_6	MK394132	326/348 (94)	1.15	-	-	1.12
A	Unidentified sp. 5671_7	MG827482	330/330 (100)	0.03	45.80	-	1.08
A	*Wickerhamomyces canadensis*	KY105899	330/337 (98)	0.59	-	-	0.58
A	*Daldinia decipiens*	LC376942	323/327 (99)	0.002	-	94.54	0.46
A	*Ogataea* sp. 5671_10	KY104428	341/344 (99)	0.45	-	-	0.44
A	Unidentified sp. 5671_13	MT535771	326/334 (98)	0.45	-	-	0.44
A	*Kuraishia molischiana*	KY103919	337/340 (99)	0.43	-	-	0.42
A	*Wickerhamomyces bisporus*	KY105897	338/345 (98)	0.23	-	-	0.22
A	*Yamadazyma scolyti*	HE612108	344/347 (99)	0.20	0.22	-	0.20
A	*Acrostalagmus luteoalbus*	MT528981	335/338 (99)	0.17	-	-	0.16
A	Unidentified sp. 5671_25	KP897542	308/309 (99)	0.007	5.18	-	0.13
A	*Fusarium* sp. 5671_18	OQ306292	319/323 (99)	0.12	-	-	0.12
A	*Lophium arboricola*	MK159395	311/316 (98)	0.007	4.17	-	0.10
A	*Scoliciosporum umbrinum*	KX133008	303/306 (99)	0.005	4.25	-	0.10
A	*Fusarium* sp. 5671_22	MT558569	303/307 (99)	0.10	-	-	0.10
			All of 20 OTUs	99.12	73.74	94.88	98.52

* A—Ascomycota, B—Basidiomycota.

## Data Availability

The relative abundance data of the related fungi presented in this study are presented in Table S1. Other data related to the study are available on request from the corresponding author.

## Supplementary Materials

The following supporting information can be downloaded at: https://www.mdpi.com/article/10.3390/insects15010049/s1. Table S1: Distribution and relative abundance of the fungal taxa directly sequenced from three species of siricid wood wasps sampled in Lithuania.
